# Quality and efficacy of Multidisciplinary Team (MDT) quality assessment tools and discussion checklists: a systematic review

**DOI:** 10.1186/s12885-022-09369-8

**Published:** 2022-03-17

**Authors:** George T. F. Brown, Hilary L. Bekker, Alastair L. Young

**Affiliations:** 1 Department of Pancreatic Surgery, St James’s University Hospital, Leeds, UK; 2 Leeds Unit of Complex Intervention Development, School of Medicine, University of Leeds, Leeds, UK; 3 Research Centre for Patient Involvement, Department of Public Health, Aarhus University, Aarhus, Denmark

**Keywords:** Cancer, Multidisciplinary team, MDT, Tumor board, Discussion, Quality assessment, Checklist, Efficacy

## Abstract

**Background:**

MDT discussion is the gold standard for cancer care in the UK. With the incidence of cancer on the rise, demand for MDT discussion is increasing. The need for efficiency, whilst maintaining high standards, is therefore clear. Paper-based MDT quality assessment tools and discussion checklists may represent a practical method of monitoring and improving MDT practice. This reviews aims to describe and appraise these tools, as well as consider their value to quality improvement.

**Methods:**

Medline, EMBASE and PsycInfo were searched using pre-defined terms. The PRISMA model was followed throughout. Studies were included if they described the development of a relevant tool, or if an element of the methodology further informed tool quality assessment. To investigate efficacy, studies using a tool as a method of quality improvement in MDT practice were also included. Study quality was appraised using the COSMIN risk of bias checklist or the Newcastle-Ottawa scale, depending on study type.

**Results:**

The search returned 7930 results. 18 studies were included. In total 7 tools were identified. Overall, methodological quality in tool development was adequate to very good for assessed aspects of validity and reliability. Clinician feedback was positive. In one study, the introduction of a discussion checklist improved MDT ability to reach a decision from 82.2 to 92.7%. Improvement was also noted in the quality of information presented and the quality of teamwork.

**Conclusions:**

Several tools for assessment and guidance of MDTs are available. Although limited, current evidence indicates sufficient rigour in their development and their potential for quality improvement.

**Trial registration:**

PROSPERO ID: CRD42021234326.

**Supplementary Information:**

The online version contains supplementary material available at 10.1186/s12885-022-09369-8.

## Background

Multidisciplinary Team (MDT) meetings are a central and mandatory part of cancer services in the United Kingdom. They are generally held on a weekly basis and are considered the gold standard for cancer care [1, 2]. Although not always obligatory, MDTs are also widely implemented internationally. Terminology varies and a cancer MDT may be alternately referred to as a tumor board meeting, multidisciplinary case review or multidisciplinary cancer conference, depending on location [3, 4]. Invariably, they are attended by a range of professionals involved in cancer management and intend to facilitate collaborative discussion between experts, with the goal of formulating timely and standardised treatment plans. This approach also aims to deliver consistently evidence-based care, provide better continuity and offer a platform for education [5]. These potential benefits have driven the growing implementation of the MDT model in global healthcare systems, against a backdrop of increasingly complex and challenging cancer treatment decisions.

It is clear that optimal MDT function, as in any clinical setting, is reliant on a multitude of factors: the availability (and distribution) of accurate clinical information, effective teamwork, appropriate attendance and strong team leadership [2, 6]. The desirable attributes of an effective MDT process have been outlined by the National Cancer Action Team (NCAT) in ‘*The Characteristics of an Effective Multidisciplinary Team (MDT)‘* [7] (Table 1). These standards are based on national survey data and incorporate the views of over 2000 MDT members [15]. They are the most widely accepted and available recommendations for MDT practice.

**Table 1:** The characteristics of an effective multidisciplinary team (MDT) [7], with comparison to domains assessed by included QATs and DCs

The characteristics of an effective MDT. Domains and subdomains		Quality Assessment Tool/Discussion Checklist					
	MDT-MODe [8]	MODe-Lite [9]	MDT-OARS [10]	MDT-MOT [11]	TEAM [12]	ATLAS [13]	MDT-QuIC [14]
**I. The Team**	✔	✔	✔	✔	✔		✔
- Membership	✔	✔			✔		✔
- Attendance	✔	✔	✔	✔	✔		
- Leadership	✔		✔	✔	✔	✔	
- Teamwork & culture	✔	✔	✔	✔	✔	✔	✔
- Personal development and training			✔	✔	✔	✔	
**II. Infrastructure**			✔	✔	✔		
- Physical environment			✔	✔	✔		
- Technology & equipment			✔	✔	✔		
**III. Meeting organisation & logistics**	✔	✔	✔	✔	✔	✔	✔
- Scheduling of meetings					✔		
- Preparation for meetings	✔	✔	✔		✔		✔
- Organisation/administration during meetings	✔		✔	✔	✔	✔	
- Post-MDT meeting/coordination of service		✔		✔	✔		
**IV. Patient-centred clinical decision-making**	✔	✔	✔	✔	✔		✔
- Who to discuss					✔		
- Patient-centred care	✔	✔	✔	✔	✔		✔
- Clinical decision-making process	✔	✔	✔	✔	✔		✔
**V. Team governance**					✔		
- Organisational support					✔		
- Data collection, analysis & audit					✔		
- Clinical governance					✔		

The evolving modern-day demographics of an aging population, increased cancer incidence and increased complexity of treatment options have resulted in a greater demand for MDT discussion, though the capacity to meet this demand remains limited [16]. Both case numbers per meeting and meeting duration have increased, whilst time per patient has conversely decreased [16, 17]. In order to manage this demand, there has been a focus on developing strategies to improve MDT efficiency, without compromising the standard of patient care. These methods may also improve consistency, by ensuring complete and standardised case presentations, as well as enabling more equal participant input.

Whilst there has been some interesting and encouraging research into the use of digital technology for decision support and case preparation [18–21], the majority of literature has so far focused on paper-based MDT quality assessment tools (QATs) and discussion checklists (DCs). Although a brief overview has previously been provided by Soukup et al. [22], the aim of this review is to provide a detailed summary of all available QATs and DCs, with a focus on assessing their development and quality. These tools can be used to measure adherence to accepted standards, as described by NCAT [7], and guide team discussions. Evidence indicating the impact tools could have in driving MDT quality improvement (QI) is also examined. The MDT in the context of this review is the cancer decision-making team specifically, but it should be recognised that forms of MDT also exist in a number of non-oncological settings, such as complex care planning or medical management.

## Methods

### Search strategy

Using OvidSP, an initial literature search was conducted of the MEDLINE, Embase and PsycInfo databases from first records until 12th November 2020. No limits were applied. Search terms were designed to reflect the various different names used to describe cancer MDTs globally. The same search was then re-run from first records until 4th January 2022 and the selection process repeated to capture any further relevant studies published in the interim period before publication.

Using the Boolean operands “AND” and “OR”, the search terms were: “MDT*” OR “multidisciplinary team* OR “multi-disciplinary team*” OR “multidisciplinary cancer conference*” OR “multi-disciplinary cancer conference*” OR “multidisciplinary case review*” OR “multi-disciplinary case review*” OR “tumour board*” OR “tumor board*” OR “tumour board meeting*” OR “tumor board meeting*” OR “tumour board review*” OR “tumor board review*” AND “proforma*” OR “pro-forma*” OR “checklist*” OR “check-list*” OR “ticklist*” OR tick-list*” OR “decision making”.

Titles were screened and duplicates removed before abstracts were scrutinised for relevance. Pertinent articles were then retrieved in full and evaluated further. Reference lists were checked for other studies of potential interest. All appropriate full-text articles were submitted for data extraction and quality appraisal.

Details of the protocol for this review were registered with the PROSPERO international prospective register of systematic reviews (PROSPERO ID CRD42021234326).

### Inclusion criteria

Full-text primary research studies were included if they described the development of a paper-based tool for the assessment of MDT process quality or guidance of discussion. Studies that used a tool for observational purposes were also selected, but only if part of the methodology could further inform the assessment of tool quality. Additionally, studies using a tool as an intervention for QI in MDT practice were also included.

Articles were not excluded based on country of origin, year of publication or language. Two researchers (GB and RR) conducted the database searches together. The same two researchers then screened titles and assessed abstracts and full-text articles for suitability independently. Any disagreements were then resolved by consensus and discussion. AY had the final decision on inclusion.

### Quality appraisal

Two researchers (GB and RR) conducted the quality appraisal process for included articles independently. Again, any disagreements were resolved by consensus and discussion, with AY having the final decision.

Methodological quality was assessed using the COnsensus-based Standards for the selection of health Measurement INstruments (COSMIN) risk of bias checklist [23]. COSMIN considers 3 main domains for study and tool quality: validity (the degree to which a tool measures what it purports to measure), reliability (the degree to which a tool is free from measurement error) and responsiveness (the ability of a tool to detect change over time). These domains are subdivided into 10 properties that may be assessed, as shown in Table 2. Each property is assessed on a 4-point scale as being very good, adequate, doubtful or inadequate. A numerical score is not assigned. As measurement tools can vary significantly, all 10 properties may not be assessed in, or relevant to, each study/tool. The COSMIN checklist is therefore a modular instrument, requiring only those properties described in the study to be appraised. Other properties are marked as not assessed.

**Table 2:** COSMIN [23] study quality appraisals

		COSMIN Risk of Bias Checklist									
		Content Validity		Internal Structure			Remaining Measurement Properties				
Study	Tool	1. Tool Development	2. Content Validity	3. Structural Validity	4. Internal Consistency	5. Cross-Cultural Validity	6. Reliability	7. Measurement Error	8. Criterion Validity	9. Construct Validity	10. Responsiveness
Lamb et al. [8]	MDT-MODe	Adequate	Very Good	N/A	N/A	N/A	Adequate	N/A	N/A	N/A	N/A
Lamb et al. [24]	MDT-MODe	N/A	N/A	N/A	N/A	N/A	Adequate	N/A	N/A	N/A	N/A
Shah et al. [25]	MDT-MODe	N/A	N/A	N/A	N/A	N/A	Adequate	N/A	N/A	N/A	N/A
Gandamihardja et al. [26]	MDT-MODe	N/A	N/A	N/A	N/A	N/A	Very Good	N/A	N/A	N/A	N/A
Jalil et al. [27]	MDT-MODe	N/A	N/A	N/A	N/A	N/A	Adequate	N/A	N/A	N/A	N/A
Soukup et al. [17]	MDT-MODe	N/A	N/A	N/A	N/A	N/A	Adequate	N/A	N/A	N/A	N/A
Soukup et al. [28]	MDT-MODe	N/A	N/A	N/A	N/A	N/A	Adequate	N/A	N/A	N/A	N/A
Soukup et al. [29]	MDT-MODe	N/A	N/A	N/A	N/A	N/A	Adequate	N/A	N/A	N/A	N/A
Hahlweg et al. al [30].	MDT-MODe	N/A	N/A	N/A	N/A	N/A	Very good	N/A	N/A	N/A	N/A
Lumenta et al. [31]	MDT-MODe	N/A	Doubtful	N/A	N/A	N/A	Very good	N/A	N/A	N/A	N/A
Lamb et al. [9]	MODe-Lite	Very Good	Very Good	N/A	Very Good	N/A	Very Good	N/A	Very Good	N/A	N/A
Taylor et al. [10]	MDT-OARS	Adequate	Doubtful	N/A	Doubtful	N/A	Adequate	N/A	N/A	N/A	N/A
Harris et al. [11]	MDT-MOT	Very Good	Very Good	N/A	N/A	N/A	Very Good	N/A	Very Good	N/A	N/A
Taylor et al. [12]	TEAM	Adequate	Very Good	N/A	Very Good	N/A	Adequate	N/A	N/A	N/A	N/A
Jalil et al. [13]	ATLAS	Very Good	Very Good	N/A	Very Good	N/A	Very Good	N/A	N/A	Very Good	N/A
Wihl et al. [32]	ATLAS	N/A	N/A	N/A	N/A	N/A	Adequate	N/A	N/A	N/A	N/A
Lamb et al. [14]	MDT-QuIC	Adequate	Very Good	N/A	N/A	N/A	N/A	N/A	N/A	N/A	N/A

Studies using a pre−/post-intervention cohort style methodology were appraised using the Newcastle-Ottawa scale for cohort studies (NOS) [33]. This assigns a score of 0–9 based on 3 domains: selection of the cohorts, comparability of the cohorts and outcome measurement. A score of 7 or more has previously been considered as representative of appropriate quality [34].

## Results

**Fig. 1:**
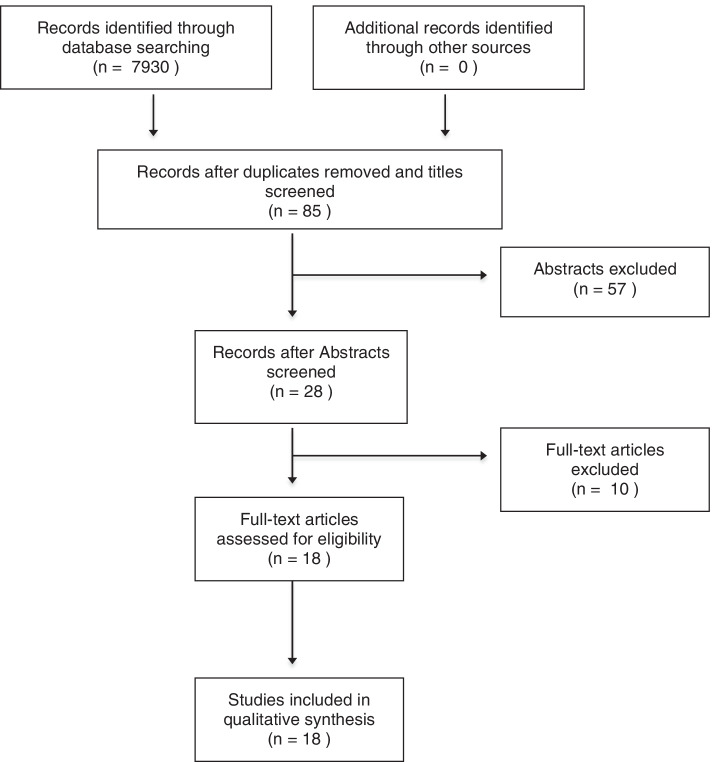
PRISMA [35] flowchart of literature search process

The final database search returned 7930 results. Titles, abstracts and finally articles-in-full were assessed using the inclusion criteria described previously. Preferred Reporting Items for Systematic Reviews and Meta-Analyses (PRISMA) [35] methodology was followed throughout (Fig. 1). 18 studies were included in the narrative data synthesis and final analysis. Data extraction was performed by 2 researchers (GB and RR) independently. Study characteristics and key results were then discussed and interpreted together with AY and HB.

### Study demographics

89% of studies were conducted in the UK and 11% were from other European centres. 7 studies described the concept, design and testing of a novel paper-based MDT QAT or DC [8–14]. 11 studies used one of these previously developed tools as part of their methodology. Of these, 5 papers were prospective and observational [25–28, 32], 3 were cross-sectional [17, 29, 30], one was a feasibility study [31] and one is best described as a cross-validation study [24]. The last included paper was a pre−/post-intervention study that used a tool for MDT QI [36]. Research was conducted in cancer MDTs of varying specialty (urology, colorectal, upper gastrointestinal, hepato-pancreato-biliary, breast, head and neck, sarcoma, skin, lung, neuro-oncology, young persons, and gynaecology).

### MDT quality assessment tools and discussion checklists

How tools compare to each other and to NCAT [7] domains of MDT process quality are shown in Table 1. Detailed descriptions of the design process and structure of each tool are presented in Additional file 1.

#### MDT-MODe

The earliest created QAT was the ‘*Metric for the Observation of Decision Making*’ (MDT-MODe). Developed by Lamb et al. [8], it was initially named the ‘*MDT Performance Assessment Tool*’ but has been referred to as MODe in most subsequent citing literature. It assesses team conduct at physical meetings and has been used to assess MDTs in real-time and via video [17, 26]. Some citing publications did make alterations to the original tool in order to be more specialty or foreign language specific [25, 30, 31]. For the purposes of this review, these studies are considered to have used MDT-MODe, as their changes did not significantly alter the tool and create a distinctively different one.

#### MODe-Lite

A recent update on the MDT-MODe [8], the MODe-Lite [9] was developed to be a shorter, more user-friendly version of the original tool for day-to-day quality assessment. Like its predecessor, it is an observational QAT and condenses the original 9 assessment domains to 6.

#### MDT-OARS

An observational QAT, the ‘*MDT Observational Assessment Rating Scale*’ [10] measures 15 areas of the MDT process across 4 main domains. These were designed to match those described in ‘*The Characteristics of an Effective Multidisciplinary Team*’ [7]. In testing, discussions were assessed in real-time and from video-recordings.

#### MDT-MOT

The ‘*MDT Meeting Observational Tool*’ [11] rates 10 domains of MDT process and is also an observational QAT. It was used to assess video-recorded MDT discussions exclusively in testing.

#### TEAM

Unlike the MDT-MODe, OARS and MOT, the ‘*Team Evaluation and Assessment Measure*’ [12] was designed for team self-assessment, rather than observation. It consists of a 47-item questionnaire, with items also directly addressing the NCAT [7] domains.

#### ATLAS

‘*A Tumour Leadership Assessment inStrument*’ [13] is distinct from other QATs, in that it specifically rates the leadership abilities of the MDT chair. The tool is, again, observational and has been used in real-time and video-recorded meetings [32].

#### MDT-QuIC

The only identified DC was the *‘MDT Quality Improvement Checklist*’. Also designed by Lamb and colleagues [14], this tool uses tick boxes to ensure there is full and appropriate discussion for each case.

### QAT/DC Role in MDT Quality Improvement

Only one study used a tool to improve MDT performance. After baseline quality assessment of a urology MDT using the MDT-MODe [8], Lamb et al. [36] introduced the MDT-QuIC [14] as part of a ‘quality improvement bundle’. The intervention also included team training and written guidance. Improvements were noted in ability to reach a decision (82.2 to 92.7%), quality of information presented (29.6 to 38.4%) and teamwork (32.9 to 41.7%). Meeting duration and time per case also reduced by 8 min and 16 s, respectively.

### Study and tool quality

COSMIN study quality appraisals are presented in Table 2. Key tool testing results are shown in Additional file 1.

After tool development, testing was generally limited to content validity, reliability and, to a lesser extent, internal consistency. The MDT-MODe [8] was the most utilised and tested QAT. Methodological quality in its design was judged to be adequate for tool development and reliability and very good for content validity. Initial testing [8] showed inter-observer agreement to be high for radiological information and contribution of oncologists, radiologists, pathologists and nurses. Intraclass correlation coefficients (ICCs) were, however, below 0.70 for all other aspects of the tool. More encouraging reliability data was provided in 9 further studies [17, 24–31]. All were considered to be methodologically adequate to very good for this property and overall inter-observer agreement was high (ICCs > 0.70). Other tools were only described in their development study or in one other citing paper. Testing results for all tools were generally supportive. ATLAS [13], MDT-MOT [11] and MODe-Lite [9] stood out in quality appraisal, scoring very good for development, content validity, reliability and internal consistency. Although not yet further studied, initial Mode-Lite [9] testing scores showed encouraging positive correlations with MDT-MODe [8] scores, indicating convergent validity. MDT-MOT [11] and MODe-Lite [9] were also rated as very good in additional testing for criterion validity and ATLAS [13] scored very good for construct validity.

All studies did, however, have some noteworthy limitations. Firstly, all tools relied on subjective human judgement. This was potentially exacerbated by the heterogeneity of observer backgrounds in testing. Secondly, observer blinding and impartiality was variable, introducing the possibility of bias. Furthermore, tools relied on direct observation, which is limited by the Hawthorne effect. Lastly, case numbers were relatively small and studies were generally single-centre, single-trust or limited to a fairly small geographical area. It is notable that the same London-based research group conducted 15 [8–14, 17, 24–29, 36] of the 18 included studies. Whilst it can be reasonably assumed that demographics here were fairly representative of the UK, this could limit tool relevance and application further afield.

Given the difference in design, the single pre−/post-intervention study [36] was appraised separately and scored 6 out of 9 on the NOS, indicating suboptimal quality (Table 3). The study’s major drawback was the lack of a comparison cohort, making any improvements more difficult to attribute definitively to the intervention. It was also reliant on the MDT-QuIC [14] and MDT-MODe [8] tools and was therefore limited by the same factors.

**Table 3:** Newcastle-Ottawa Scale [33] Study Appraisals

		Newcastle-Ottawa Quality Assessment Scale								
		Selection				Comparability	Outcome			Total
Study	Tool	1.	2.	3.	4.	1.	1.	2.	3.	
Lamb et al. [36]	MDT-QuIC	★	★	★	–	- / -	★	★	★	6

## Discussion

This is the first review to systematically investigate paper-based MDT QATs and DCs and enables clinical teams to identify and compare tool characteristics and make informed decisions. These tools can be used to monitor performance in line with NCAT [7] standards. Evidence to suggest tool benefit in MDT QI is described. It is, however, envisaged that identification of their shortcomings will be of more benefit, identifying areas for more specific research and aiding the development of other tools in future.

Most QATs focused on assessing aspects of physical meetings, such as case information, leadership, attendance and teamwork. Governance, infrastructure and logistical elements of the MDT process were less frequently addressed. There were options for team self-assessment as well as observation. All QATs used Likert scales to assess each domain, with corresponding descriptions of optimal to suboptimal practice. There were no objective outcome measures. As they were used in isolation, the limitations of Likert scales should be considered [37]. One DC (MDT-QuIC [14]) was identified.

Although testing was usually limited to certain properties of validity and reliability, methodological quality in tool design was generally adequate. The concept and development of each tool was evidence-based and addressed some, if not all, of NCAT [7] MDT quality domains. Tools were considered acceptable and clinician feedback was positive. Additionally, their simple nature makes them cost-effective and easily introduced.

Importantly, a single study, using the MDT-QuIC [14] as part of a ‘quality improvement bundle’, did demonstrate a positive real-world impact on MDT discussion [36]. These results are encouraging, but are far from definitive - especially given the study’s limitations and mixed methods intervention. The paucity of studies using these tools for QI is reflective of the fact that, to date, they have mainly been utilised in observational research as the measure of quality, rather than the stimulus. This is an important distinction and highlights a significant void in the literature. These tools reasonably claim to be a method of identifying areas for improvement, but so far there is little evidence to substantiate this claim. A considerable amount of further research is required to better investigate their efficacy in QI. Given the nature of MDT discussion, randomised controlled trials are unlikely to be feasible, but controlled studies with QAT/DC-specific exposures would be beneficial to better demonstrate their role in creating change rather than simply measuring it.

Significantly, what these studies did not address was the effect tools had on the quality of the treatment decision itself. Tool domains closely reflect NCAT [7] standards and, as such, they are compared to those in this review. It is important to understand, however, that these guidelines focus very much on the MDT process, rather than on what constitutes quality in the actual discussions and their outcomes. This raises the question of what ‘quality’ these tools are assessing and guiding towards. Clearly, an effective process is desirable, but correct and reproducible decisions will always be the most important indicator of MDT value.

Specific interest in discussion quality itself is growing, with some evidence suggesting that performance in this area is not always optimal [38–41]. Discussions tend to be dominated by biomedical information and led by surgeons and other diagnosticians [39, 41]. Nurse specialist and other allied health professional input is more likely to be marginalised, ignored or non-existent [38, 42]. These traditional hierarchies are potentially damaging, as unequal contribution defeats the purpose of collective expertise and opinion. Lanceley et al. [39] also demonstrated the human nature of MDT discussion, highlighting the influence of personal experience and ethics. The potential for bias and groupthink in team decision-making is well known [43] and MDTs are not excluded from this.

These factors could be extremely damaging to the MDT model, based as it is on the principle is that collective experience and decision-making is superior to single clinician lead care. Survey data suggests that clinicians are widely in agreement that MDT discussion is beneficial, but high-quality evidence to prove this beyond doubt remains elusive [44]. Equally, there is limited data to evidence whether survival is truly improved by MDT discussion [45, 46]. In their systematic review, Lamb et al. [38] showed that MDT discussion did alter treatment decisions, but studies generally failed to correlate these changes with actual improvement in patient outcomes. Given the potential problems of team decision-making, this lack of definitive evidence certainly challenges the steadfast authority of the MDT within cancer services, as well as questioning their economic cost. Indeed, one study found a single MDT could cost up to £10,050 every month [47]. Ultimately, the tools presented in this review do not adequately assess MDT discussion and decision quality specifically or sufficiently enough to fully address these concerns. Once again, further investigation is required and future research should focus on ways to reliably assess discussions themselves and investigate effects on patient outcomes, rather than process quality alone.

Finally, the ‘unknown quantity’ in MDT decisions is patient choice. Autonomy is central to ethical healthcare and the importance of shared decision-making is enshrined in *‘Good Medical Practice*’ [48]. Notably, 6 [8–12, 14] of the 7 tools identified in this review did incorporate scoring for (indirect) discussion of patient views. However, given their scope, this remained a small part of the overall assessment. Currently, patient involvement in MDT decisions does appear to be limited [38]. Tellingly, one study found only 4% of investigated MDTs directly involved patients in their own treatment discussion [49]. Interestingly, evidence suggests that nurses are more likely to advocate for patients in the decision-making process [22, 38], further reinforcing the importance of equal participation. In light of the apparent barriers to patient involvement, calls to review the process have been made [ 50. ]. Could the nurse specialist have a bigger role in the discussion by proactively presenting the patient’s views? Or should the MDT outcome be a range of options that are then presented to the patient in clinic? What is clear is that greater integration of psychosocial factors will only add to the complexity of treatment decisions, making consistency and structural solidity even more essential. Tools aiding standardisation in the process may therefore have a greater role in the MDT of the future.

As MDTs evolve, digital solutions are also likely be utilised more frequently. Of these, decision support systems [18, 19, 51] may be particularly advantageous, as they offer rapid integration of patient information with evidence-based guidelines to generate objective management options. Early evidence has shown that this can increase guideline compliance and appropriate trial recruitment [18]. Going forwards, a combination of tools and technologies could be used to achieve the goal of high standards and reproducibility in MDT decision-making.

In summary, this review identifies and presents several paper-based tools for assessing the MDT process and guiding team discussions. Methodological quality was generally acceptable. These tools were developed to measure against, and increase compliance with, accepted high standards in the general MDT process. They represent a practical and relatively simple intervention that teams could employ to monitor their performance according to those standards and potentially identify areas for improvement. Extremely limited and relatively poor quality evidence supports the use of one tool in facilitating elements of MDT QI. Whether these tools overall could have a positive impact on decision quality and, crucially, on patient outcomes has not been established.

## Supplementary Information

**Additional file 1.** Characteristics, methodology and key results of included studies [52, 53].

## Data Availability

All data generated or analysed during this study are included in this published article (and its supplementary information files).

## Abbreviations

MDT: Multidisciplinary team
NCAT: National cancer action team
QAT: Quality assessment tool
DC: Discussion checklist
QI: Quality improvement
COSMIN: Consensus-based standards for the selection of health measurement instruments
NOS: Newcastle-Ottawa scale
PRISMA: Preferred reporting items for systematic reviews and meta-analyses
MDT-MODe: MDT metric for observation of decision-making
MODe-Lite: Metric for observation of decision-making (short version)
MDT-OARS: MDT observational assessment rating scale
MDT-MOT: MDT meeting observational tool
TEAM: Team evaluation and assessment measure
ATLAS: A tumour leadership assessment measure
MDT-QuIC: MDT quality improvement checklist
ICCs: Intraclass correlation coefficients
