# Supplementary material for: Quality and efficacy of Multidisciplinary Team (MDT) quality assessment tools and discussion checklists: a systematic review
Source: BMC Cancer. 2022 Mar 17;22:286. doi: 10.1186/s12885-022-09369-8 (PMC8928609; doi:10.1186/s12885-022-09369-8)
Supplement: Supplementary file 1 — Additional file 1. Characteristics, methodology and key results of included studies [52, 53]. [file 12885_2022_9369_MOESM1_ESM.docx]

| **Author, Year**  **Additional file 1.** Characteristics, methodology and key results of included studies | **Country** | **Study Design** | **Tool** | **Participants** | **Methodology** | **Key Results** |
| --- | --- | --- | --- | --- | --- | --- |
| Lamb et al. (2011)^22^ | United Kingdom | Concept, design, testing | MDT-MODe | 3 cancer MDTs, 112 case discussions | Tool developed from literature search and modification of the ‘*Observational Teamwork Assessment for Surgery*’ tool^52^, which measures behaviors in the operating theatre. The MDT-MODe was adapted for assessing MDT discussions and is scored on a case-by-case basis. The tool rates different aspects of MDT activity under the two broader domains of clinical information quality and teamwork. 9 areas of MDT case discussion were rated on 5-point Likert scales, with 5 being optimal and 1 being poor practice. The areas scored were: case history, radiological information, pathological information, leadership of the MDT chairperson and contribution of the surgery, radiology, pathology, oncology and nurse specialist attendees.  Expert MDT members checked the tool for face validity.  2 Independent observers (surgeon & psychologist) rated MDT performance using the tool. | Inter-observer correlation = ICC >0.70 for presentation of radiological  Information, contribution of oncologists,  radiologists, pathologists and clinical nurse specialist. ICCs = 0.68 for case history, 0.69 for surgeon contribution, 0.52 for chairperson contribution, 0.31 for pathology. |
| Lamb et al. (2011)^35^ | United Kingdom | Cross-validation | MDT-MODe | 5 cancer MDTs, 164 cases | Team members self evaluated their MDT function on an online survey. The MDT was observed by an independent observer using the MTD-MODe tool. The self and observer assessments were then compared. | Overall positive correlation between self and observer assessment. Highest correlation in case history and radiology. Lowest in patient-centered metrics. Median Spearman’s rho = 0.74. |
| Shah et al. (2014)^28^ | United Kingdom | Prospective, observational | MDT-MODe | Colorectal MDT, 267 case discussions | Independent observation of MDT and rating using assessment tool MDT-MODe, modified for to be colorectal specific. | Good inter-observer reliability for tool with different levels of experience. Overall ICC = 0.79. |
| Gandamihardja et al. (2019)^29^ | United Kingdom | Prospective, observational | MDT-MODe | Breast MDT, 346 case discussions | Independent observation of an MDT using the tool. | Inter-observer correlation coefficents overall = >0.70 (0.73-0.93). |
| Jalil et al. (2014)^30^ | United Kingdom | Prospective, observational | MDT-MODe | Urology MDTs at 4 sites. 556 case discussions rated in person, 127 rated from video | 2 surgical residents were trained to use the assessment tool. They used this to rate MDT’s in person and from watching videos of MDT’s. | Good inter-observer reliability after 40-60 cases for both in-person and video assessments (ICC >0.70) and ‘mainly’ from then on. |
| Lumenta et al. (2019)^34^ | Austria | Feasibility | MDT-MODe | 4 cancer MDTs, 244 case discussions | Literature reviewed and MDT-MODe tool adapted by researchers for German speakers. MDT’s were observed by 3 independent observers using the tool. | The percent agreement between the different pairs of raters was mostly similar and generally high. |
| Soukup et al. (2020)^10^ | United Kingdom | Cross-sectional | MDT-MODe | 3 cancer MDTs, 822 case discussions | Used tool as one method to assess MDT team performance. Calculated ICC for inter-observer agreement. | Inter-observer agreement ICC = 0.934. |
| Soukup et al (2018)^31^ | United Kingdom | Prospective, observational | MDT-MODe | Breast MDT, 1335 case discussions | Used tool to assess MDT function to assess if teams suffered from decision-making fatigue. Did calculate inter-observer agreement. | Phase 1: r = 0.89  Phase 2: r = 0.92 |
| Soukup et al (2016)^33^ | United Kingdom | Cross-sectional | MDT-MODe | 4 cancer MDTs, 1045 case discussions | Used MDT-MODe tool to assess MDT decision-making. Did calculate inter-observer agreement. | ICC for each team = 0.92, 0.83, 0.86, 0.71. |
| Hahlweg et al. (2017)^32^ | Germany | Cross-sectional | MDT-MODe | 11 MDTs, 249 cases | Used MDT-MODe, modified for German language, to assess MDT decision-making. Did calculate inter-observer agreement. | Cohen’s Kappa/ICC ≥ .5 was reached for all independent variables, except for quality of radiological information, quality of information on comorbidities and quality of information on the patient views. |
| Lamb et al. (2021)^26^ | United Kingdom | Concept, design, testing | MODe-Lite | 6 MDTs, 146 case discussions | 2 meetings were held between experts to design an abridged version of the original MDT-MODe. The tool scored 6 domains of MDT case discussion: clinical input, holistic input, radiology, pathology, clinical collaboration, and management plan. Each domain was scored on a 3-point scale for inclusion of 1 = none, 2 = partial or 3 = all of the tool’s recommended information for each domain. In phase 2, a single author used the tool to assess 146 cases previously scored using the original MDT-MODe and MeDiC^53^ tool. Scores were compared to assess convergent and external validity. In phase 3 a further 60 cases were scored by 2 assessors using the tool to assess interobserver agreement. | MODe-Lite domain scores had positive correlations with the global score and the global scores of the original MDT-MODe and MeDiC^53^ scores, supporting hypothesis 1 & 2 . Pathology was the only domain that did not reach statistical significance. Cronbach alpha scores were good for pathology, radiology and management plan. Scores were weaker for clinical input, holistic input and clinical collaboration. ICC’s were generally above 0.7. |
| Taylor et al. (2012)^23^ | United Kingdom | Concept, design, testing | MDT-OARS | 10 bowel cancer MDTs | Tool developed from literature search and expert panel.  The tool measures 15 areas of MDT function across 4 main domains: Team, infrastructure, organisation and logistics and clinical decision-making. Areas are rated using 4-point Likert scales, with 1 being very poor and 4 being very good.  MDT’s were assessed in vivo and via video by independent observers then interviews were conducted with MDT members. | Stated that presence of camera and observer in MDT acceptable and didn’t change behavior.  Stated that some internal consistency was observed.  ICCs of at least 0.50 (and up to 0.92) were achieved for all but 2 characteristics - the absence  of tension/conflict (ICC = 0.10) and evidence of treatment planning (ICC = 0.32). |
| Harris et al. (2016)^25^ | United Kingdom | Concept, design, testing | MDT-MOT | 10 cancer MDTs | MDT-MOT tool developed using literature and views of 20 MDT’s. It was then piloted and reviewed by an expert panel.  The tool assessed 10 areas with each rated on a 5-point Likert scale, 1 being suboptimal and 5 being optimal practice. These areas were: attendance, leadership and chairing, team work and culture, development and training, physical environment, venue, technology and equipment, organisation and administration, patient-centered care, decision-making and post-meeting co-ordination of service.  13 novice healthcare workers used the tool to (1) assess video excerpts from a model MDT and a poor quality MDT (2) evaluate 10 video recorded MDT’s which were also assessed by 2 expert observers. | Criterion validity = assessors were able to differentiate between a good and poor MDT (p = <0.005).  Inter-rater reliability = Weighted Kappa = >0.60 for clinical decision-making, organisation and administration during MDT meetings and leadership of the MDT and chairing.  In these 3 areas, plus patient-centered care, at least 80% of observers’ ratings were in absolute agreement |
| Taylor et al. (2012)^20^ | United Kingdom | Concept, design, testing | TEAM | 23 cancer MDTs completed questionnaire, plus interview of 74 members.  10 cancer MDTs also completed questionnaires to test content validity | Tool developed by expert panel working from data supplied by 60 MDT’s. Created a 47 item self-assessment questionnaire. Each item is in the form of a statement (e.g., *“leadership could be improved in our team’*). Each statement is responded to using a 5-point Likert scale, with 1 being completely disagree and 5 being completely agree.  Items cover 5 domains, 17 subdomains and exactly match NCAT’s ‘*Characteristics of an Effective Multidisciplinary Team*’. | Content validity = responses to the 47-item questionnaire were strongly and significantly correlated with responses to the domain-specific questionnaires  (Pearson’s correlation coefficent = 0.67, p = <0.01).  Internal consistency = leadership, teamwork and culture had a Cronbach alpha of >0.70, with a further five domains achieving alpha ratings >0.60. Other items were lower.  Item discrimination = 4 items had a moderate correlation of r = 0.60–0.65 but the majority had little correlation.  Acceptability & feasibility = Overall positive views. |
| Jalil et al. (2018)^21^ | United Kingdom | Concept, design, testing | ATLAS | 114 MDT members for content validation. 17 MDT leaders, 417 case discussions | ATLAS tool was developed using literature and expert input to specifically assess MDT *leadership*. The tool consisted of 12 domains rated on a 5 point Likert scale: Time management, communication, allowing contribution, ability to summarise, clear treatment plan, prioritisation, managing distraction, facilitating discussion, managing conflict, leadership, atmosphere, recruiting to trials.  The tool was presented to MDT members who completed a survey. 2 expert observers then used the tool assess MDT leaders in vivo and via video. Comparison with the MDT-MODe was used for hypothesis testing. | Content validity = All above 0.78.  Internal consistency = Cronbach’s alpha = 0.80.  Inter-observer reliability = ICCs 0.65-0.88 for video assessment, 0.68-0.91 in vivo.  Construct validity = correlation between ATLAS and MODe for chairing scores (r = 0.617, p = 0.014). There was a non-significant correlation between ATLAS and MODe for other parameters (r= −0.196, p = 0.483). |
| Wihl et al. (2020)^27^ | Sweden | Prospective, observational | ATLAS | 3 cancer MDTs observed 10-12 times | Independent observation of MDT meetings using ATLAS tool. Observations compared. | Inter-observer agreement ICC = 0.72 for entire sample set.  Stated easy to use. |
| Lamb et al. (2012)^24^ | United Kingdom | Concept, design, testing | MDT-QuIC | 175 MDT members | MDT-QuIC checklist developed using literature and expert panel.  The checklist consists of tick boxes under 4 main domains: before case discussion (core members present?, somebody present who knows the patient?, is the patient’s key worder present?), information (case history, comorbidities, radiological, pathological, psychosocial, patient’s views, clinical trials, other), discussion (surgeons, physicians, oncologists, radiologists, pathologists, nurses, palliative care, allied professionals) and outcome (what are the recommendations of the MDT?, are there any objections?, is further discussion required?).  MDT members completed a survey after viewing the tool. | Median responses to all but one question about the checklist were significantly positive (p = <0.001).  Respondents thought it could be used prepare cases for MDT meetings, to structure case discussions, or as a record of MDT discussion. Most thought it should be used by the MDT chair |
| Lamb et al. (2013)^36^ | United Kingdom | Pre/post-intervention | MDT-QuIC | 36 urology MDT meetings. 1421 case discussions over 16 months | Baseline data collected for 2 months before 4 part intervention phases. These were half day team training, introduction of MDT-QuIC for self-assessment, resident lead MDT-QuIC and written guidance. Outcome measures were whether a treatment decision was made and recorded and whether this was related to improved teamwork. MDT’s were assessed for teamwork by an independent assessor using the MDT-MODe tool. | Overall improvement in ability to reach a treatment decision over time (82.2% to 92.7%), improved quality of information presentation (29.6% to 38.4%) and improved teamwork (32.9% to 41.7%).  Ability to reach a treatment decision was related to the quality of available information (r = 0.298; p = <0.05) and quality of teamwork within the MTB (r = 0.348; p = < 0.05).  Meeting time and time per reduced. |
